# Circulating androgen receptor combined with 18F-fluorocholine PET/CT metabolic activity and outcome to androgen receptor signalling-directed therapies in castration-resistant prostate cancer

**DOI:** 10.1038/s41598-017-15928-y

**Published:** 2017-11-14

**Authors:** V. Conteduca, E. Scarpi, P. Caroli, S. Salvi, C. Lolli, S. L. Burgio, C. Menna, G. Schepisi, S. Testoni, G. Gurioli, G. Paganelli, V. Casadio, F. Matteucci, U. De Giorgi

**Affiliations:** 1 Department of Medical Oncology, Istituto Scientifico Romagnolo per lo Studio e la Cura dei Tumori (IRST) IRCCS, via Maroncelli 40, 47014 Meldola, Italy; 2 Unit of Biostatistics and Clinical Trials, Istituto Scientifico Romagnolo per lo Studio e la Cura dei Tumori (IRST) IRCCS, Meldola, Italy; 3 Nuclear Medicine Operative Unit, Istituto Scientifico Romagnolo per lo Studio e la Cura dei Tumori (IRST) IRCCS, Meldola, Italy; 4 Biosciences Laboratory, Istituto Scientifico Romagnolo per lo Studio e la Cura dei Tumori (IRST) IRCCS, Meldola, Italy

## Abstract

The association between choline uptake and androgen receptor (*AR*) expression is suggested by the upregulation of choline kinase-alpha in prostate cancer. Recently, detection of *AR* aberration in cell-free DNA as well as early 18F-fluorocholine positron emission tomography/computed tomography (FCH-PET/CT) were associated with outcome in metastatic castration-resistant prostate cancer (mCRPC) patients treated with abiraterone and enzalutamide. We aimed to make a direct comparison between circulating *AR* copy number (CN) and choline uptake at FCH-PET/CT. We analysed 80 mCRPC patients progressing after docetaxel treated with abiraterone (n = 47) or enzalutamide (n = 33). We analysed *AR* CN from plasma samples using digital PCR and Taqman CN assays and total lesion activity (TLA) and metabolic tumor volume (MTV) on FCH-PET/CT at baseline. A meaningful correlation was showed among *AR* gain and TLA/MTV compared to *AR* non-gained cases (P = 0.001 and P = 0.004, respectively), independently from type of treatment. Multivariate analysis revealed that *AR* CN and only TLA were associated with both shorter PFS (P < 0.0009 and P = 0.026, respectively) and OS (P < 0.031 and P = 0.039, respectively). AR gain appeared significantly correlated with choline uptake represented mainly by TLA. Further prospective studies are warranted to better address this pathway of AR-signalling and to identify multiplex biomarker strategies including plasma *AR* and FCH-PET/CT in mCRPC patients.

## Introduction

Prostate cancer (PC) is the most common cancer type for the estimated new cancer cases and the third tumour for the estimated deaths among men in 2015 worldwide^1^. Androgen receptor (AR) signalling axis is the most important actor in PC pathogenesis and in the progression to castration resistant PC (CRPC), representing a fundamental target of androgen deprivation therapy^2^. In PC, multiple direct mechanisms can stimulate AR signalling and often lead to therapeutic resistance, including *AR* copy number (CN) gain or somatic point mutations^3–6^ and constitutively active AR splice variants such as AR-V7^7^. Among the indirect mechanisms responsible of increased AR protein expression, there is also the up-regulation of choline kinase alpha (CHKA). It binds directly the AR ligand-binding domain (LBD), fulfilling different roles involved in the tumour growth and disease progression^8,9^. Classical function of CHKA in Kennedy pathway is the production of phosphatidylcholine, essential for plasma membrane biogenesis^10^. In addition, CHKA has a “nonclassical” function, interacting with cytoplasmatic AR and promote its stability. CHKA plays a role as a chaperone for AR, resulting in the ubiquitination and activation of AR–dependent transcription. Recent genome wide approaches^11^ suggested this dual interaction between CHKA and AR in cytoplasm, promoting AR overexpression and increased signalling, which in turn may provide more CHKA production.

Some studies have documented the overexpression of CHKA protein in multiple cancer types including breast, colorectal, endometrial, lung, ovarian, and prostate, providing an increased uptake of choline^12–14^.

Choline is a relatively new radiopharmaceutical for positron emission tomography (PET)/computed tomography (CT) imaging, and its utility in visualizing and staging different tumours has been published^15–18^, especially PC^19–22^, by mainly using 18F-fluorocholine (FCH), its radiopharmaceutical analogue.

Recent studies, investigating the early response assessment on FCH-PET/TC imaging^21,22^ in metastatic CRPC (mCRPC) patients treated with novel anti-AR therapies, such as abiraterone^23^ and enzalutamide^24^, have strengthened the hypothesis of a relationship between choline uptake and AR.

In this study, we evaluated the choline uptake in mCRPC patients measuring the total lesion activity (TLA) and metabolic tumour volume (MTV) which have shown much greater prognostic value than conventional PET measurements such as the maximum standardized uptake value (SUVmax)^25,26^. We aimed to identify an association between circulating *AR* CN and choline uptake to better understand the complex AR signalling axis and alternative pathways of the resistance mechanisms, such as choline metabolism, and to improve multiplex biomarker strategy in mCRPC patients.

## Results

### Patient characteristics

Between October 2011 and November 2015, 80 mCRPC patients (47 treated with abiraterone and 33 with enzalutamide) were enrolled for this study. Overall median age was 74 years (range 42–90). All patients previously received a docetaxel-based regimen, and 35 (43.8%) patients received previous treatments [15 (31.9%) before abiraterone and 20 (60.6%) before enzalutamide, P = 0.02]. At baseline, all patients had evidence of mCRPC and were categorized according to the extent of metastases in a binary categorical variable, defined as bone or lymph node only (low spread) including 40 cases, and visceral involvement or combined bone and lymph node disease (extensive spread) including 40 cases without differences between abiraterone and enzalutamide group. On the contrary, the median number of metastatic lesions on FCH-PET/TC is significant different between two groups [10 (range 1–36) and 18 (range 1–47) for abiraterone and enzalutamide, respectively, P = 0.03]. Androgen receptor was gained in 24 cases (30%) with no significant difference in *AR* gain prevalence in the two treatment groups. Table 1 summarizes patient characteristics.

**Table 1:** Patient characteristics.

	TOTAL	Abiraterone	**Enzalutamide**	**p**
	(N = 80)	(N = 47)	**(N** = **33)**	
	N (%)	N (%)	**N (%)**	
**Age** (years)	74	72	74	
median value (range)	(42–90)	(57–90)	(42–90)	0.201
**Baseline PSA, ng/mL**	45.59	32.0	65.0	
median value (range)	(0.2–4351)	(0.2–1229)	(2.24–4351)	0.045
**Baseline TLA**	393430.8	394517.0	387626.7	
median value (range)	(11429–3543821))	(11429–3529614)	(11755–3543821)	0.853
**Baseline MTV**	112.25	99.1	128.86	
median value (range)	(2.15–787.19)	(2.66–787.19)	(2.15–567.40)	0.374
**FCH PET/TC lesions, N**	13	10	18	
median value (range)	(1–47)	(1–36)	(1–47)	0.030
**ECOG PS, N (%)**				
0–1	77 (96.2)	44 (93.6)	33 (100)	
2	3 (3.8)	3 (6.4)	0	0.264
**Gleason score, N (%)**				
6 – 7	29 (42.0)	17 (41.5)	12 (42.9)	
≥ 8	40 (58.0)	24 (58.5)	16 (57.1)	0.909
**Previous therapeutic lines, N (%)**				
1	45 (56.2)	32 (68.1)	13 (39.4)	
≥ 2	35 (43.8)	15 (31.9)	20 (60.6)	0.020
**Cell**–**free** ***AR*** **CN, N (%)**				
Normal	56 (70.0)	33 (70.2)	23 (69.7)	
Gain	24 (30.0)	14 (29.8)	10 (30.3)	0.961
**Baseline extent of metastasis, N (%)**				
Bone or lymph	40 (50.0)	23 (48.9)	17 (51.5)	
Bone + lymph or visceral	40 (50.0)	24 (51.1)	16 (48.5)	0.821

*Abbreviations*. AR, androgen receptor; CN, copy number; ECOG, Eastern Cooperative Oncology Group; FCH-PET/CT, 18F-fluorocholine positron emission tomography/computed tomography; MTV, metabolic tumor volume; N, number; PS, performance status; PSA, prostate-specific antigen; TLA, total lesion activity.

### Correlation between *AR* status and choline uptake

**Figure 1:**
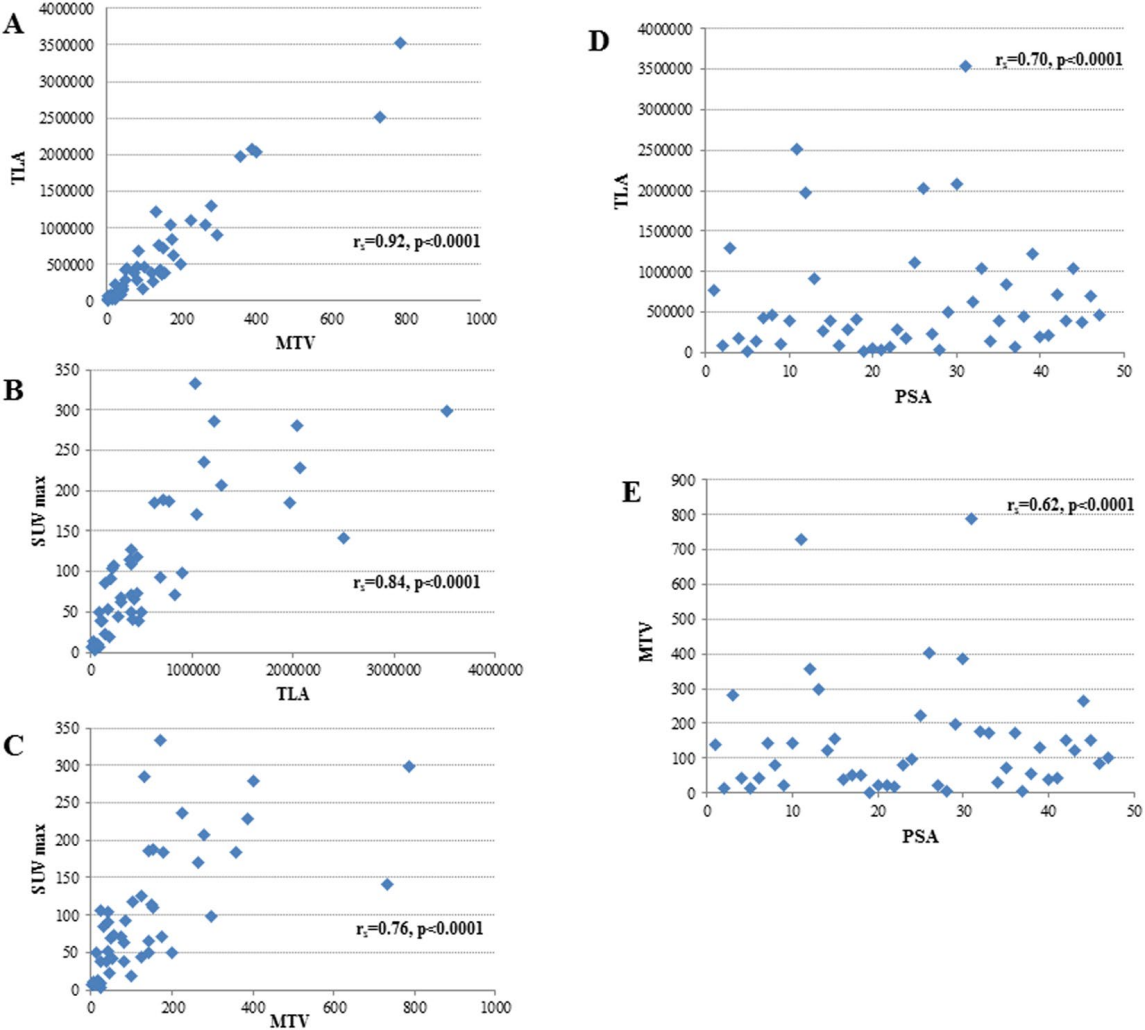
Correlation among different FCH-PET/CT parameters and baseline PSA levels to demonstrate the reliability of presented data.

**Figure 2:**
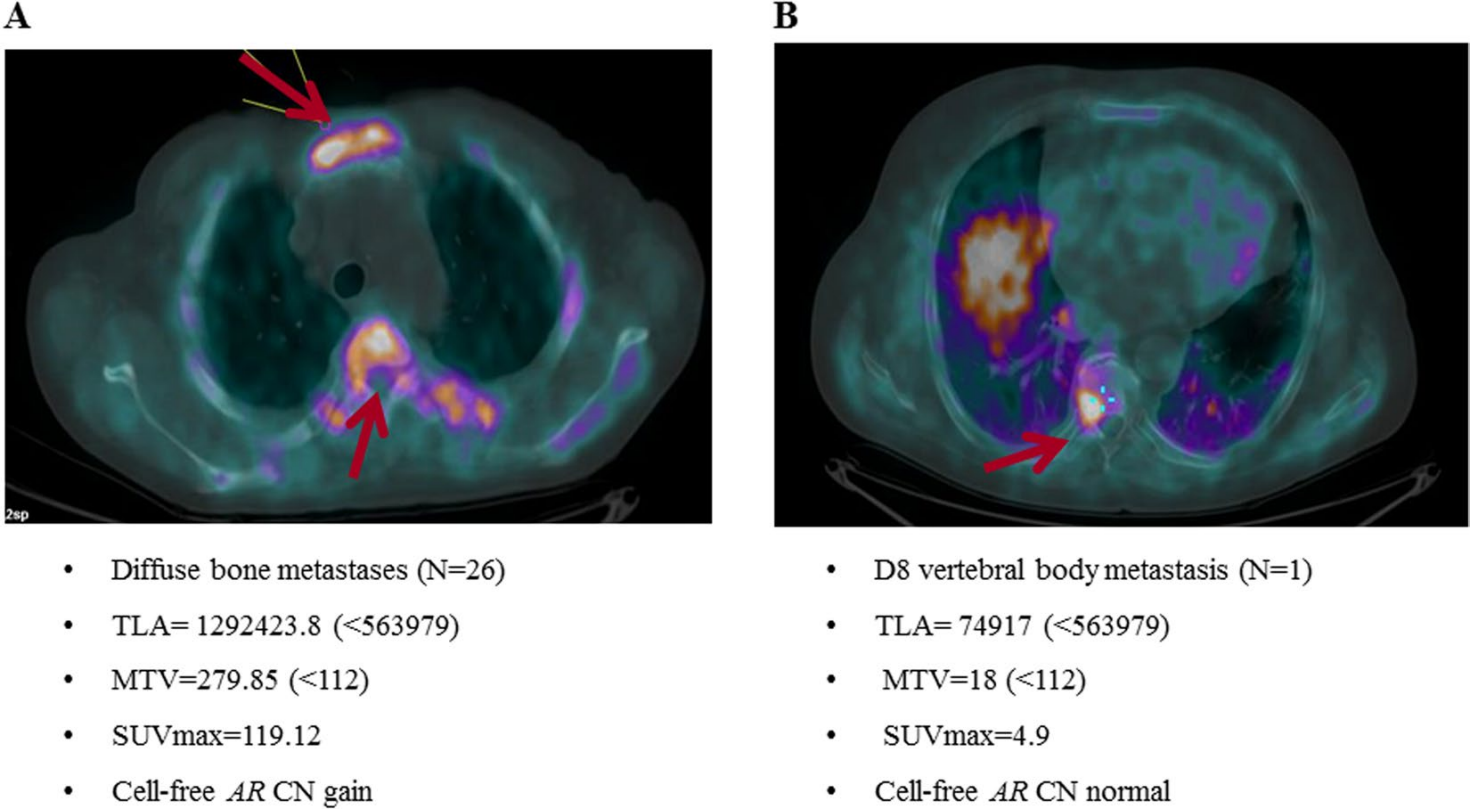
FCH-PET/CT of two metastatic CRPC patients. The FCH PET/CT image (A) demonstrates diffuse metabolically active skeletal metastases involving multiple vertebrae (arrows) with high values of TLA, MTV, SUVmax in one *AR*-gained patient. The FCH PET/CT image (**B**) shows involving one dorsal vertebrae (arrows) with high low values of TLA, MTV, SUVmax in one *AR*-normal patient.

The reliability of the presented data has been shown in the Fig. 1, comparing median TLA and MTV values with standardized uptake value maximum (SUVmax) and prostate specific antigen (PSA) level. The ROC curves for the best cut-off value for TLA and MTV identified 563,979 and 112, respectively. Thirty-one (38.7%) and 40 (50%) patients had TLA and MTV above the cut-off value, respectively. We showed a strong association between high values of TLA and MTV and cell-free *AR* gain (P = 0.005 and P = 0.004, respectively) (Table 2). Figure 2 represented this correlation showing two different cases treated with abiraterone and characterized by high choline uptake/*AR* CN gain (A) and low choline uptake/*AR* CN normal (B), respectively.

**Table 2:** Association among TLA, MTV and cell-free *AR* status.

	**Cell-free** ***AR*** **CN**		**p**
	**Normal**	**Gain**	
	**N (%)**	**N (%)**	
**Baseline TLA** ^*^			
<563979	40 (71.4)	9 (37.5)	
≥563979	16 (28.6)	15 (62.5)	0.005
**Baseline MTV** ^*^			
<112	34 (60.7)	6 (25.0)	
≥112	22 (39.3)	18 (75.0)	0.004

^*^Determined by ROC curves.

*Abbreviations*. *AR*, androgen receptor; CN, copy number; MTV, metabolic tumor volume;.

N, number; TLA, total lesion activity.

### Ability of *AR* status and choline uptake to predict survival

At the time of analysis, 77 (96.2%) out of 80 patients had progressive disease (PD) and 51 (63.7%) patients had died. Median follow-up was 22 months (range 1–42). The median progression-free survival (PFS) and overall survival (OS) were 6.4 months [95% confidence interval (CI) 3.8–7.5) and 15.4 months (95% CI 11.0–22.4), respectively].

**Figure 3:**
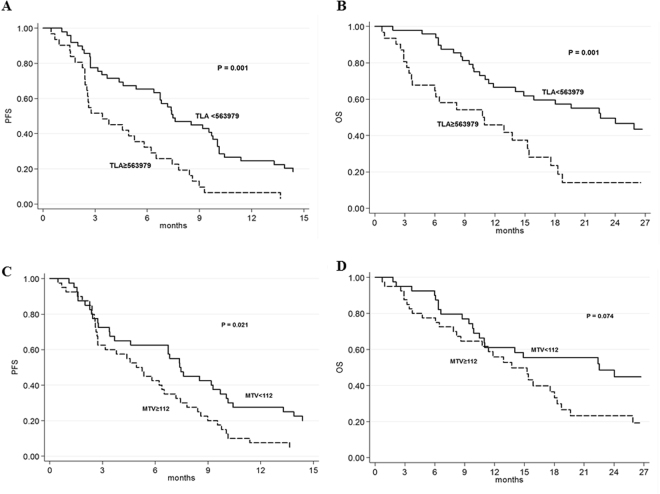
Association of choline uptake with outcome. Progression free survival (**A**) and overall survival (**B**) according to total lesion activity and progression free survival (**C**) and overall survival (**D**) according to metabolic tumor volume.

Univariate and multivariate Cox proportional hazards regression analyses were performed to assess the associations between circulating *AR* CN and FCH-PET/CT and outcome. In the univariate analysis, *AR* status, all PET parameters, and number of metastatic lesions predicted significantly PFS and OS (Table 3). In CRPC patients with baseline high values of TLA and MTV determined by using ROC curves, Kaplan–Meier curves demonstrated a shorter PFS (3.4 vs 7.5 months, P = 0.001, and 5.1 vs 7.4 months, P = 0.021, respectively) and OS (11.0 vs 22.5 months, P = 0.0009, and 13.7 vs 22.5 months, P = 0.074, respectively) (Fig. 3). We performed multivariate analysis adjusted for age, TLA, MTV, cell-free *AR* CN, number and site of metastatic lesions, and number of previous therapeutic lines. Only high TLA and cell-free *AR* gain resulted significantly associated with worse PFS [hazard ratio (HR) 2.03 (95% CI 1.09-3.78; p = 0.026), and HR 2.68 (95% CI 1.49-4.82; p = 0.0009), respectively] and OS [HR 2.41 (95% CI 1.04-5.55; p = 0.039), and HR 2.09 (95% CI 1.07-4.10, P = 0.031), respectively] (Table 4).

**Table 3:** Univariate analysis of progression-free survival and overall survival.

	N patients	N events	Median PFS (months) (95% CI)	P	N events	Median OS (months) (95% CI)	P
**Overall**	80	77	6.4 (3.8–7.5)	—	51	15.4 (11.0–22.4)	—
**Age** ^**§**^							
<74	39	37	5.3 (2.6–9.0)		24	13.7 (8.6–29.9)	
≥74	41	40	6.7 (3.4–7.8)	0.795	27	18.0 (11.4–24.0)	0.840
**TLA** ^*^							
<563979	49	46	7.5 (6.4–9.8)		28	22.5 (14.0–39.8)	
≥563979	31	31	3.4 (2.4–5.8)	0.001	23	11.0 (3.7–15.4)	0.0009
**MTV** ^*^							
<112^1^	40	37	7.4 (3.7–9.7)		23	22.5 (10.5–39.8)	
≥112	40	40	5.1 (2.7–6.5)	0.021	28	13.7 (8.6–18.0)	0.074
**Cell**–**free** ***AR*** **CN**							
Normal	56	53	7.4 (5.8–9.2)		34	18.7 (11.8–29.9)	
Gain	24	24	2.9 (2.3–5.3)	0.0001	17	10.8 (6.0–15.9)	0.007
**ECOG PS**							
0–1	77	74	6.4 (3.8–7.4)		49	15.9 (11.0–22.5)	
2	3	3	8.4 (0.5–11.4)	0.879	2	12.9 (11.4–nr)	0.811
**Gleason score**							
<8	29	29	7.5 (3.8–9.3)		20	14.9 (9.9–25.9)	
≥8	40	38	4.1 (2.6–6.7)	0.998	25	15.3 (7.9–22.4)	0.990
**N. of previous therapeutic lines**							
1	45	43	6.8 (3.4–8.4)		27	18.7 (11.8–28.3)	
>1	35	34	4.9 (2.8–7.4)	0.448	24	11.4 (8.2–18.3)	0.242
**Site of metastatic lesions**							
Bone or lymph	40	38	6.6 (3.4–8.4)		22	15.9 (11.0–nr)	
Bone + lymph or visceral	40	39	6.1 (2.7–7.8)	0.444	29	13.7 (9.8–24.0)	0.345
**N. of metastatic lesions** ^**§**^							
≤20	50	47	7.1 (4.6–9.7)		28	22.5 (11.0–39.8)	
>20	30	30	4.5 (2.5–7.4)	0.002	23	13.7 (6.4–17.6)	0.005

^*^Cutoff determined by ROC curve.

^§^Cutoff determined by median value.

*Abbreviations*. *AR*, androgen receptor; CI, confidence interval; CN, copy number; ECOG, Eastern Cooperative Oncology Group; MTV, metabolic tumor volume; N, number; OS, overall survival; PFS, progression-free urvival; PS, performance status; TLA, total lesion activity.

**Table 4:** Multivariable Cox Proportional Hazard Analysis of Predictors of Progression-free Survival and Overall Survival.

	PFS		OS	
	HR (95% CI)	P	HR (95% CI)	P
**Age (continuous variable)**	0.98 (0.96-1.01)	0.291	0.99 (0.96-1.02)	0.633
**TLA** ^*^				
<563979	1.00		1.00	
≥563979	2.03 (1.09-3.78)	0.026	2.41 (1.04-5.55)	0.039
**MTV** ^*^				
<112	1.00		1.00	
≥112	0.92 (0.48-1.79)	0.815	0.75 (0.32-1.78)	0.519
**Cell-free** ***AR*** **CN**				
Normal	1.00		1.00	
Gain	2.68 (1.49-4.82)	0.0009	2.09 (1.07-4.10)	0.031
**N. of metastatic lesions** ^§^				
≤20	1.00		1.00	
>20	1.19 (0.61-2.32)	0.615	1.13 (0.45-2.87)	0.790
**Site of metastatic lesions**				
Bone or lymph	1.00		1.00	
Bone + lymph or visceral	1.44 (0.91-2.29)	0.119	1.31 (0.74-2.32)	0.356
**N. of previous therapeutic lines**				
1	1.00		1.00	
>1	1.25 (0.79-1.98)	0.346	1.48 (0.83-2.62)	0.184

^*^Cutoff determined by ROC curve.

^§^Cutoff determined by median value.

*Abbreviations*. *AR*, androgen receptor; CI, confidence interval; CN, copy number; MTV, metabolic tumor volume; N, number; OS, overall survival; PFS, progression-free survival; TLA, total lesion activity.

No significant association between MTV, TLA, and PSA response rate has been observed, as well as for FCH PET/TC response (Supplementary Table S1).

## Discussion

Our results confirm the indisputable role of *AR status* in guiding the treatment decision and monitoring clinical outcome of mCRPC patients treated AR signalling-directed therapies as abiraterone and enzalutamide. In addition, we showed the importance of alternative pathways associated with AR signalling. Recently, the challenge of molecular stratification of cancer patient through liquid biopsy using circulating tumor DNA^27^ has allowed qualifying plasma *AR* as a minimally invasive genetic biomarker of clinical outcome and resistance to abiraterone or enzalutamide^3^. The baseline association between cell-free *AR* gain and choline uptake has clearly and firstly emerged from the results of this paper in CRPC patients treated with abiraterone and enzalutamide.

In the last decade, some studies attempted to explore the relationship between FCH-PET/TC and other possible surrogate as predictive and prognostic factors, such as Gleason score, PSA kinetics, PSA trigger, PSA velocity, PSA double timing, circulating free-DNA levels, in both localized and metastatic prostate tumor and in relation to different treatments^28–32^.

In our study, we evidenced the utility of FCH-PET/CT parameters as potential no-invasive prognostic factors in CRPC patients. However, we assessed no significant correlation among TLA and MTV values and the site and number of metastases, probably since the overall choline uptake may be affected not only by total tumor burden but also by tumor metabolic activity and other known factors, such as the number of viable cells per unit volume and the tumour vascularization^33^ and, in some cases, emergence of neuroendocrine differentiation, especially secondary to androgen deprivation therapy^34^.

In addition, we observed no relationship among *AR* CN, TLA/MTV and PSA levels, likely because the peculiar action mechanisms of abiraterone and enzalutamide can often lead to an unusual PSA modulation^35,36^.

The metabolic tumor features, represented by TLA and MTV, and their clinical impact may make possible new ways of managing and investigating mCRPC. Currently, the use of FCH-PET/TC is still controversial as an imaging tool in clinical practice, mainly because of its costs, diagnostic accuracy and discomfort for additional imaging examinations for the patient. However, there is a significant amount of FCH-PET/CT data published showing a high degree of good accuracy for PC detection, also comparing to a standard imaging exam, such as magnetic resonance^37,38^.

Clinical and pre-clinical studies have already pursued CHKA as a therapeutic target in patients with solid tumors^11,39^. Because of identifying specific relationship between increased choline uptake and plasma *AR* gain, our work can lead to future phase I and II trials in PC considering to use CHKA inhibitors in patient with *AR* amplification to overcome possible mechanism of resistance. Particularly, as CHKA binds directly the AR LBD, we could select for the treatment with CHKA inhibitors only the patients with a full-length *AR* mutated in the LBD and/or gain of *AR* and exclude the limited subset of patients expressing only AR variants such as AR-V7 because they lack AR LBD and could not interact with CHKA.

A strength of this study is the performance of ddPCR to assess *AR* CN in our samples that is less expensive and time-consuming than next generation sequencing methods but with a high agreement with NGS data, as previously demonstrated^3^.

The main limitations of this study were its sample size, retrospective design and single institution setting. All PET/CT indices are no absolute measures, which can be affected by factors such as PET scanner calibration, image reconstruction method and acquisition, the timing of tracer administration. Consequently, parameters from this study may not necessarily be optimal for other institutions. Moreover, larger and prospective studies are warranted to validate these data using a relatively common threshold-based method [e.g. cut-off by ROC curves in our study or by median values in Kwee’s paper^28^ to define a standardized score prognostic of functional imaging in combination with other factors. In addition, the post-transcriptional regulation of *AR* by CHKA leads to consider *AR* CN change precedes by far the choline uptake phenomenon. Consequently, future studies should discover more *AR* downstream signalling events and so identify molecules as post-translational modifiers of *AR* in association with CHKA.

In conclusion, this study firstly combined two biomarker approaches of imaging and genomics may implement the patient treatment selection, avoiding useless overtreatment and toxicity. This work increases the prospects for personalized CRPC treatment by stratifying patient cohorts according to their *AR* copy number and FCH-PET/CT parameters. Additional genomic alterations such as *AR* point somatic mutations and/or splice variants could be correlated with FCH-PET/CT to obtain a stronger combination between molecular features and functional imaging. However, it needs a further evaluation and validation with a large scale study and a prospective design of study, including also chemotherapy-naive population. Lastly, this study provided strong supportive evidence for the prognostic role of functional imaging. So we also could investigate molecular data in association with PET/CT with other new tracers, such as radiolabelled dihydrotestosterone and radiolabelled antibody to prostate-specific membrane antigen (PSMA)^40–42^, in order to better define tumour biology, mechanism of treatment resistance and potential therapeutic targets in mCRPC patients.

## Methods

### Study design

Eighty consecutive patients with mCRPC without neuroendocrine differentiation in progression after docetaxel and treated with abiraterone and enzalutamide were included in this retrospective study. Selection criteria included histological confirmation of adenocarcinoma of the prostate, baseline serum testosterone <50 ng/dL, Eastern Cooperative Oncology Group (ECOG) performance status ≤2, ongoing androgen deprivation therapy. Exclusion criteria were renal insufficiency and/or concomitant therapy with proton pump inhibitors which could influence the CgA levels and so potential onset of neuroendocrine differentiation. All patients had completed previous chemotherapy at least 4 weeks before to allow baseline examinations for abiraterone and enzalutamide to be performed. Metastatic disease was documented by bone scan, computed tomography or magnetic resonance imaging. In the group of patients treated with abiraterone, therapy consisted of 28-day cycles of daily abiraterone acetate 1000 mg with twice-daily prednisone 5 mg. In the group of patients treated with enzalutamide, treatment consisted of enzalutamide 160 mg once daily. The choice of therapy was at the discretion of the treating physician. In each therapy group, treatment was continued until there was evidence of PD or unacceptable toxicity. Peripheral blood samples for plasma DNA extraction for the investigation of cell-free *AR* CNs and FCH-PET/CT imaging studies were obtained within 30 days of therapy initiation. During treatment, PSA response and toxicity were evaluated on a monthly basis. FCH-PET/CT was repeated after 3 to 6 weeks. A CT scan was performed after 3 months of abiraterone and enzalutamide therapy. Disease progression was defined according to Prostate Cancer Working Group 2 (PCWG2) criteria^43^. The study included patients participating in a protocol approved by the Institutional Review Board of Istituto Scientifico Romagnolo per lo Studio e la Cura dei Tumori (IRST), Meldola, Italy (REC 2192/2013). The study was approved by the Local Ethics Committee (Ethics Committee Area Vasta Romagna and IRST) and informed consent for the use of biological material for research purposes was obtained from all patients before plasma sample collection. All experiments were performed in accordance with relevant guidelines and regulations.

### PET/CT Imaging Protocol

FCH-PET/CT scans were performed on an integrated PET/CT system (Discovery LS camera, General Electric Medical Systems, Waukesha, WI) in 2D acquisition mode for 3 minutes per bed position. PET scanning was performed 45 minutes after intravenous injection of 18F-Methilcholine (3,7 MBq/Kg of body weight, AAA-Advanced Accelerator Applications, Meldola, Italy). The field of view comprised the skull to mid-femurs. Low dose CT (120 kV, 80 mA) without contrast agents was carried out for attenuation correction and as an anatomical map. The emission data were adjusted for scatter, random coincidence events, and system dead time utilizing the provided software.

The reading and interpretation of PET/CT scans were assigned to two nuclear medicine physicians with consolidate experience. Criteria to define PET/CT positivity included the presence of focal areas of increased tracer uptake with or without any underlying lesion identified using CT. Semiquantitative criteria based on SUVmax and the target to background ratio were used to aid the visual analysis^44^. Each patient had the MTV calculated as the sum of each three- dimensional volumes of interest. Moreover, for each lesion volume and SUV mean was multiplied and then summed to generate TLA. The first estimate MTV is a purely volumetric entity, while TLA also takes the metabolic activity of the lesion into account, thereby producing an estimate of the tumour activity^45^. FCH-PET/CT images were read sequentially by using a Xeleris III workstation (General Electric Medical Systems). PET, CT, and PET/CT fused images provide to properly assess the scans in axial, sagittal, and coronal cuts.

### Detecting copy number gain of AR in plasma

Peripheral blood samples were collected within 30 days of each treatment initiation, maintained at room temperature, processed within 30 min, and stored at −80 °C. Circulating DNA was extracted from 1 to 2 ml of plasma with the QIAamp Circulating Nucleic Acid Kit (Qiagen) according to the manufacturer’s instructions. DNA quality and concentration were estimated using spectrophotometry quantification (NanoDrop ND-1000, Celbio, Milan, Italy). We performed copy number of androgen receptor (AR) by duplex TaqMan quantitative real-time PCR (qPCR) assay (Applied Biosystems, Foster City, CA) and multiplex droplet digital PCR on a QX200 ddPCR system (Bio-Rad). *AR* CN assays were performed using AR gene and at least two different reference genes: *RNaseP*, *NSUN3*, *ElF2C1*, and *AP3B1* and *ZXDB* at Xp11.21 as a control gene, as previously described^3–5^.

### Statistical analysis

Progression-free survival was defined as the time between the first day of abiraterone and enzalutamide treatment and the date of PD or death (whichever came first). Patients who had not progressed at database closure were censored at the last tumour evaluation or discontinuation of treatment due to toxicity. Overall survival was defined as the time between the first day of treatment and the date of death from any cause or the date of the last follow-up visit. Receiver-operating characteristics (ROC) analysis was performed to estimate accuracy of TLA and MTV, considered as continuous variables. The area under the ROC curve with 95% CI was calculated. The comparison of MTV and TLA median values in patients with or without *AR* gain was performed using median test (Wilcoxon). PFS and OS were estimated using the Kaplan-Meier method and survival curves were compared using the logrank test. Cox regression model was used to investigate potential predictors of PFS and OS and to estimate HR and their 95% CI. All P-values were two-sided and a P < 0.05 was considered as statistically significant. Statistical analyses were performed with SAS 9.4 software (SAS Institute, Cary, NC, USA).

Results of this study have been presented in part at the European Society of Medical Oncology (ESMO) Cancer Congress 2015 in Vienna, Austria, 25 September–29 September, 2015 (poster presentation session).

## Electronic supplementary material

Supplementary Table S1
