# Supplementary material for: Circulating androgen receptor combined with 18F-fluorocholine PET/CT metabolic activity and outcome to androgen receptor signalling-directed therapies in castration-resistant prostate cancer
Source: Sci Rep. 2017 Nov 14;7:15541. doi: 10.1038/s41598-017-15928-y (PMC5686214; doi:10.1038/s41598-017-15928-y)
Supplement: Supplementary file 1 — Supplementary Table S1 [file 41598_2017_15928_MOESM1_ESM.pdf]

Circulating androgen receptor combined with 18F-fluorocholine PET/CT metabolic activity and outcome to androgen receptor signalling-directed therapies in castration-resistant prostate cancer

V. Conteduca[1]*, E. Scarpi[2], P. Caroli[3], S. Salvi[4], C. Lolli[1], S. L. Burgio[1], C. Menna[1], G. Schepisi[1], S. Testoni[2], G. Gurioli[4], G. Paganelli[3], V. Casadio[4], F. Matteucci[3], U. De Giorgi[1]

[1]Department of Medical Oncology, Istituto Scientifico Romagnolo per lo Studio e la Cura dei Tumori (IRST) IRCCS, via Maroncelli 40, 47014 Meldola, Italy
[2]Unit of Biostatistics and Clinical Trials, Istituto Scientifico Romagnolo per lo Studio e la Cura dei Tumori (IRST) IRCCS, Meldola, Italy
[3]Nuclear Medicine Operative Unit, Istituto Scientifico Romagnolo per lo Studio e la Cura dei Tumori (IRST) IRCCS, Meldola, Italy
[4]Biosciences Laboratory, Istituto Scientifico Romagnolo per lo Studio e la Cura dei Tumori (IRST) IRCCS, Meldola, Italy

Supplementary Table S1. PSA Response Rate according to *AR* copy number and TLA/MTV

	**PSA Response Rate**		
	**No**	**Yes**	
	**No. (%)**	**No. (%)**	**p**
AR N & TLA <563979	10 (43.5)	6 (40.0)	
AR N & TLA ≥563979	6 (26.1)	2 (13.3)	
AR A & TLA <563979	1 (4.3)	4 (26.7)	
AR A & TLA ≥563979	6 (26.1)	3 (20.0)	0.282
AR N & MTV <112	9 (39.1)	6 (31.6)	
AR N & MTV ≥112	7 (30.4)	6 (31.6)	
AR A & MTV <112	1 (4.4)	3 (15.8)	
AR A & MTV ≥112	6 (26.1)	4 (21.0)	0.528

Abbreviations: A, amplified; *AR*, androgen receptor; CI, confidence interval; MTV, metabolic tumor volume; N, normal; TLA, total lesion activity.
